# Pharmacist interventions to improve hypertension management among patients with diabetes: a systematic review and meta-analysis of randomized controlled trials

**DOI:** 10.1186/s12913-025-13461-7

**Published:** 2025-10-01

**Authors:** Viktoria Gastens, Stefano Tancredi, Dylan Bonnan, Blanche Kiszio, Cinzia Del Giovane, Ross T. Tsuyuki, Gilles Paradis, Arnaud Chiolero, Line Guénette, Valérie Santschi

**Affiliations:** 1 Population Health Laboratory (#PopHealthLab), University of Fribourg, Fribourg, Switzerland; 2 La Source, School of Nursing Sciences, HES-SO University of Applied Sciences and Arts Western Switzerland, Lausanne, Switzerland; 3 Population Health and Optimal Health Practices Research Unit, CHU de Québec Research Centre, Faculty of Pharmacy, Université Laval, Québec City, Québec Canada; 4 Institute of Primary Health Care (BIHAM), University of Bern, Bern, Switzerland; 5 Department of Medical and Surgical Sciences for Children and Adults, University-Hospital of Modena and Reggio Emilia, Modena, Italy; 6 EPICORE, Department of Medicine, University of Alberta, Edmonton, Canada; 7 School of Population and Global Health, McGill University, Montreal, Canada

**Keywords:** Cardiovascular health, Hypertension, Team-based care, Pharmaceutical care, Systematic review

## Abstract

**Background:**

Improving blood pressure (BP) control is of major importance among patients with diabetes, due to their high risk of micro- and macrovascular complications. Community-based models of care with the involvement of pharmacists and other nonphysician healthcare providers can help manage hypertension. We aimed to estimate the effectiveness of pharmacist interventions, alone or in collaboration, on BP among outpatients with diabetes and hypertension.

**Methods:**

We conducted systematic searches of randomized controlled trials (RCTs) assessing the effect of pharmacist interventions on BP among outpatients with hypertension and diabetes compared to usual care. The outcome was the systolic and diastolic BP change or BP control. We performed a meta-analysis with a random effects model to estimate mean differences in BP or relative risk of BP control, with 95% confidence intervals (CIs). The protocol was registered in PROSPERO (CRD42021279751) and published in an open-access peer-reviewed journal.

**Results:**

Out of 2330 study records identified by electronic database searching, we included 12 studies in the systematic review, with 5,256 participants (control 2,906; intervention 2,350), published between 2002 and 2022. In comparison with usual care, mean systolic and diastolic BP were reduced after pharmacist interventions by -7.2 mmHg (95% CI: -12.5 to -1.9; I^2^ = 87%) and − 4.1 mmHg (95% CI: -6.9 to -1.4; I^2^ = 0%), respectively. BP control was improved after pharmacist intervention (relative risk: 1.76, 95% CI: 1.39 to 2.24; I^2^ = 50%). Analyses restricted to relatively large or high-quality studies yielded consistent but slightly lower estimates.

**Conclusion:**

Pharmacist interventions improve BP control in patients with diabetes and hypertension.

**PROSPERO registration number:**

CRD42021279751.

**Supplementary Information:**

The online version contains supplementary material available at 10.1186/s12913-025-13461-7.

## Background

Diabetes increases the risk of developing cardiovascular and chronic kidney diseases. A meta-analysis of 102 prospective studies involving 698,782 individuals found that diabetes confers about a 2-fold increased risk for a wide range of vascular diseases, such as coronary heart disease, stroke, and deaths from cardiovascular causes, including heart failure, cardiac arrhythmia, sudden death, hypertensive disease, and aortic aneurysms, independently from other conventional risk factors [1]. The risk for complications is even higher when diabetes coexists with hypertension. Framingham data showed that the population with hypertension at the time of diabetes diagnosis had higher rates of all-cause mortality (32 vs. 20 per 1000 person-years) and cardiovascular events (52 vs. 31 per 1000 person-years) compared to normotensive patients with diabetes, suggesting that much of the excess risk is attributable to coexisting hypertension [2]. 

Diabetes and hypertension share common causes and risk factors. Factors involved in the pathogenesis of both hypertension and diabetes include inappropriate activation of the renin-angiotensin-aldosterone system, oxidative stress, inflammation, impaired insulin-mediated vasodilatation, augmented sympathetic nervous system activation, altered innate and adaptive immunity, and abnormal sodium processing by the kidney [3]. Patients with diabetes experience increased peripheral artery resistance caused by vascular remodeling and increased body fluid volume associated with insulin resistance-induced hyperinsulinemia and hyperglycemia. Both mechanisms elevate systemic blood pressure (BP) [4]. 

Improving BP control is therefore of major importance among patients with diabetes to decrease the high risk of micro- and macrovascular complications. Team-based models of care with the involvement of pharmacists and other nonphysician healthcare providers can help manage hypertension [5, 6]. The United States Community Preventive Services Task Force, and more recently the American College of Cardiology, the American Heart Association, and the European Societies of Cardiology and Hypertension all recommend team-based care in hypertension management, with the involvement of pharmacists [7–10]. Indeed, pharmacists have a pivotal role as the most accessible healthcare providers and experts in medication management. For instance, they can provide patient education, e.g., to improve health behaviours, and help patients adhere to medications.

Several meta-analyses of randomized trials have shown that pharmacist interventions, either pharmacist-led or in collaboration with other healthcare professionals, can help decrease BP [5, 11]. A meta-analysis published in 2012 found that pharmacist interventions improve major cardiovascular disease risk factors (including BP, cholesterol, and body mass index) among outpatients with diabetes [12]. This systematic review applied less strict inclusion criteria (i.e., including patients with diabetes, whether or not they had hypertension) and did not include outcomes important for hypertension and diabetes management such as BP control or hemoglobin A1c (HbA1c) levels. Additionally, more recent synthesized evidence is missing. We aimed, therefore, to systematically review and synthesize evidence of the effect of pharmacist interventions, alone or in collaboration, on BP among outpatients with diabetes and hypertension.

## Methods

We followed the Cochrane Collaboration and Centre for Reviews and Dissemination guidance methods for conducting and reporting systematic reviews and meta-analyses [13, 14] and reported the results of this review according to the Preferred Reporting Items for Systematic Reviews and Meta-Analyses statement (PRISMA) [15]. This study is a subgroup analysis of a systematic review assessing the effect of pharmacist interventions on BP in outpatients with hypertension, for which the protocol was registered on the International Prospective Register of Systematic Reviews (PROSPERO) database (CRD42021279751) and published in an open-access peer-reviewed journal [16]. 

### Eligibility criteria

Inclusion and exclusion study criteria were based on specific (1) study designs, (2) settings, (3) participants, (4) interventions, (5) comparators, and (6) outcomes. Randomized controlled trials (RCTs), cluster RCTs, and cross-over RCTs were eligible. Case reports, case series, non-randomized evaluations, reviews, meta-analyses, conference proceedings, policy papers, study protocols, and expert opinions were excluded. Studies performed in a community/ambulatory care setting were included. Studies were considered if they included adult outpatients (18 years or over) with diagnoses of hypertension and diabetes, treated by medication or not, and if they evaluated the effect of pharmacist-led or collaborative interventions in outpatients compared to usual care. We distinguished between outpatient clinics (hospital or medical facility without overnight stay) and community care (e.g., a community pharmacy or general practitioner). Outcomes of interest were the mean difference between intervention and usual care group in BP change from baseline to follow-up or BP control (BP below a predefined target level) at follow-up. We considered all publications in English, French, and German and searched all databases from inception to the date of search.

### Information sources

We searched the following electronic databases: MEDLINE (Ovid) (from 1946 on), Excerpta Medica database (Embase) (from 1947 on), Cochrane Central Register of Controlled Trials (CENTRAL) (from 1947 on), Cochrane Database of Systematic Reviews (CDSR) (from 1995 on), CINAHL (EBSCO) (from 1937 on), Web of Science (from 1900 on), JBI EBP Database (Ovid) (from 1998 on), and Tripdatabase (from 1997 on). The search for unpublished studies included the Grey Literature Report (New York Academy of Medicine, www.greylit.org). The search was conducted on 26.03.2024.

### Search strategy

The specific search strategies (Table S1) were developed by an experienced medical librarian in systematic review searches (BK) in consultation with the project team. They were constructed to include the two main concepts of this systematic review: ‘hypertension’ and ‘pharmacist intervention’. A three-step search strategy was used in this review. First, an initial limited search of MEDLINE (Ovid) was undertaken using the search terms ‘Pharmacist intervention’, ‘Pharmacists’, ‘Pharmaceutical Services’, ‘Pharmacy Service, Hospital’, ‘Pharmacies’, ‘Pharmacy’, ‘Hypertension’, ‘Blood pressure’. Second, an analysis of the text words contained in each article’s title, abstract, and index terms was undertaken to expand the list of search terms. Based on the results of this analysis, a more thorough search was conducted in the chosen databases. The search strategy for MEDLINE was created first and was then adapted for each database, including all identified keywords and index terms. Third, the reference lists of all included studies selected for critical appraisal were searched by hand and cited reference searches for all included studies were conducted in Web of Science to find any additional studies not identified during the initial search processes. The methodology search filter to limit retrieval to appropriate study designs, a modified version of the Cochrane Highly Sensitive Search Strategy, was used to identify randomized trials [13]. 

### Selection process

Study records retrieved by electronic searching were uploaded to the systematic review management software Covidence [17]. We performed (1) a systematic review of pharmacists’ interventions in hypertension management [16], and (2) subgroup analyses focusing only on patients with both hypertension and diabetes. After removing duplicates, titles and abstracts were independently screened by two reviewers (VG and ST) for inclusion. The reviewers indicated whether a citation was potentially relevant (met inclusion criteria), clearly not relevant (met exclusion criteria), or if the information was insufficient to make a judgment. We obtained full-text publications for all titles/abstracts that appeared to meet inclusion criteria or where there was any uncertainty. Full-text publications were independently examined by two reviewers (VG and ST) to select studies for inclusion. Reasons for excluding ineligible studies were recorded. Any disagreement was resolved through discussion and, if required, by consulting a third review author (AC or VS). Studies included in the primary systematic review focusing on pharmacist interventions in outpatients with hypertension were further screened by two reviewers (VG and DB) for meeting the inclusion criteria concerning a diabetes diagnosis. The selection process was recorded in detail in a Preferred Reporting Items for Systematic Reviews and Meta-Analyses (PRISMA) flow diagram [15]. 

### Data collection process

Data extraction was conducted using a prespecified data extraction template in the systematic review management software Covidence [17]. Two reviewers (VG and ST) independently extracted the data listed in Table S2 from each eligible study. Outcomes related to diabetes (e.g., HbA1c) were extracted by two independent reviewers (VG and DB).

The primary outcome data extracted was the mean difference between intervention and control groups in systolic and diastolic BP change from baseline to follow-up and the corresponding standard error (continuous outcome). If not reported in studies, the mean difference in BP change and standard error were calculated from reported information (BP change per study group, BP at baseline, BP at follow-up, and standard deviation (SD), corresponding confidence intervals or p values of subgroup analyses). If no BP change or BP baseline information was available, the mean difference between intervention and control groups at follow-up was used [18]. For BP control (dichotomous, secondary outcome), we extracted the proportion of participants reaching a pre-defined BP target level. The target BP could differ from one study to the other. For outcomes related to diabetes, we extracted the mean HbA1c change between baseline and follow-up, mean HbA1c and SD at baseline and follow-up, HbA1c control (% reaching a pre-defined target) at baseline and follow-up, glucose (mg/dl) at baseline and follow-up, and the corresponding standard errors.

We classified pharmacist interventions using the following pre-defined categories: (1) pharmacist-led (i.e., initiated and managed solely by a pharmacist) and (2) in collaboration (i.e., the pharmacist is involved in interventions together with a multidisciplinary healthcare team) [19]. Based on the Cochrane Effective Practice and Organization of Care (EPOC) taxonomy interventions, we categorized pharmacist interventions by different recipients (patient, healthcare provider) and types (educational approach, e.g., targeted toward patients to improve their lifestyle; feedback, e.g., to healthcare providers to adapt medication; use of reminder tools, e.g., drug adherence aids) [20]. 

### Risk of bias assessment

Two reviewers (VG and ST) independently assessed the risk of bias for each study using the ‘Cochrane Risk of Bias Tool’ for randomized trials (RoB 2) [21]. This tool assesses the risk of bias according to the following domains: (1) Randomization process; (2) Effect of assignment to intervention; (3) Missing outcome data; (4) Measurement of the outcome; (5) Selection of the reported result. The risk of bias assessment for cluster-randomized trials and randomized crossover trials was performed with the specific RoB 2 tools for these study designs [21]. 

We classified the risk of bias for each domain as either ‘Low risk’, ‘Some concerns’ or ‘High risk’ and provided information from each study together with the reasons for our evaluation [13, 21]. Given the type of RCTs included in our review, blinding the participants and the research teams was usually not feasible; only the outcome assessment could be blinded. The quality of BP measurement was also systematically assessed along three criteria: (1) use of clinically validated BP measurement devices; (2) training of outcome assessor; (3) measurement of BP out of the office. We derived an overall study risk of bias as follows: ‘Low risk’ if all domains were at low risk of bias, ‘Some concerns’ if one domain was of some concerns, and ‘High risk’ if at least one domain was at high risk of bias or multiple domains were judged with some concerns in a way that substantially lowers confidence in the result [21]. We resolved any disagreements in quality assessment through discussions and the involvement of an arbitrator (AC or VS) when necessary.

### Effect measure

For the continuous outcome, we used the mean difference in BP change between groups whenever available. When this measure was not reported, we used the mean difference in BP between groups at the end of the follow-up [18]. Calculations for the between-group standard error were based on calculations provided in the Cochrane Handbook [13]. The pooled effect was calculated as weighted mean differences in BP between intervention and usual care groups, with 95% confidence intervals (CIs). For the dichotomous outcome of BP control, we calculated the relative risk as the ratio of the proportion of BP control (%) at follow-up in the pharmacist intervention group versus the usual care group and estimated the pooled relative risk (RR) comparing intervention versus usual care groups, with 95% CI.

### Statistical analyses

All analyses were conducted with R 4.2.2 (The R Foundation) using the meta software package [22]. A random effects model was used to estimate the pooled effects and results are displayed in forest plots. Between-studies heterogeneity was quantified using the I^2^ statistic. To assess the robustness of our results, we performed sensitivity analyses among studies of high quality and larger size. Publication bias was evaluated by visual inspection of funnel plots, and funnel plot asymmetry was examined using the Egger test [13, 23]. The Grading of Recommendations Assessment, Development and Evaluation (GRADE) framework was applied to assess the strength of the body of evidence for this systematic review [24]. Domains used to determine the certainty of the evidence included risk of bias, inconsistency, indirectness, imprecision, and publication bias. The certainty of evidence was assessed for systolic BP by including all studies and then excluding studies with a high risk of bias.

## Results

### Study selection and characteristics

**Fig. 1:**
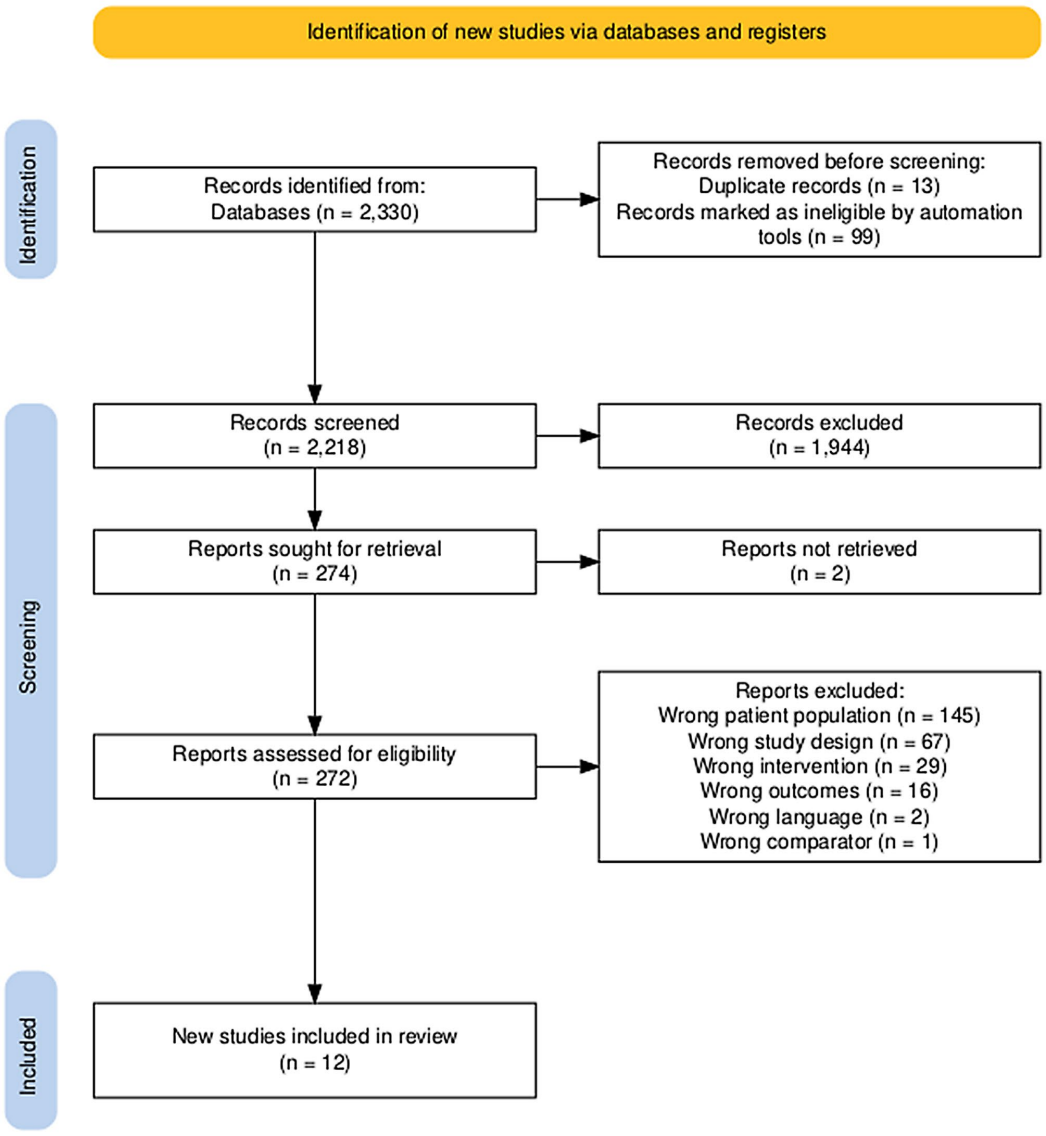
Flow diagram of studies assessed and included [47]

A total of 2330 study records were identified by electronic database searching and loaded into the systematic review management software Covidence [17]. After removing duplicates, 2218 studies were independently screened based on titles and abstracts by two authors, 274 full texts were evaluated for eligibility (Fig. 1), and 95 studies were included in the primary systematic review of pharmacist interventions’ effect on BP among patients with hypertension [16]. After screening these studies for patients with diabetes, a total of 12 studies, all published in the English language, were included in our review [25–36], including 5,256 participants (control 2,906; intervention 2,350). The characteristics of each study are presented in Table S3. Reasons for exclusion of studies (*n* = 260) were most commonly related to an inappropriate patient population, including the absence of diabetes (*n* = 145), study design (*n* = 67), and intervention (*n* = 29) [37–39]. 

A summary of studies and patient characteristics is shown in Table 1. Included studies were published between 2002 and 2022 and conducted in different regions (United States: *n* = 7, Canada: *n* = 1, Mexico: *n* = 1, Indonesia: *n* = 1, China: *n* = 1, Pakistan: *n* = 1). The characteristics of the pharmacist interventions are summarized in Table 2. In 67% of the studies, the intervention was led by the pharmacist and in 33%, they collaborated with other healthcare providers. Interventions targeted patients in 83% of the studies and healthcare providers in 67%. The types of interventions comprised patient education in 83%, healthcare provider education in 33%, feedback to healthcare providers (e.g., medication change) in 42%, and patient reminders (e.g., drug adherence aids) in 8% of the studies, respectively. Feedback to healthcare providers included medication change, either after independent pharmacist prescribing or recommendations to physicians. Further details on the characteristics of studies and pharmacist interventions are displayed in Table S3.

**Table 1:** Summary of study and patient characteristics of the included studies

Study characteristics	*N* = 12
Region	
Pan-American	9 (75%)
European	0 (0%)
Western Pacific	1 (8%)
South-East Asian	1 (8%)
Eastern Mediterranean	1 (8%)
African	0 (0%)
Setting	
Outpatient clinics	9 (75%)
Community pharmacies	3 (25%)
Duration of follow-up in months	
Mean (min, max)	6 (3, 9)
Outcome	
BP change	5 (42%)
BP at follow-up	2 (17%)
BP control	7 (58%)
**Patient characteristics**	
Mean age	
Mean (min, max)	60 (42, 69)

**Table 2:** Summary of pharmacist intervention characteristics of the included studies

Intervention characteristics	*N* = 12
Pharmacist care	
Pharmacist-led	8 (67%)
Pharmacist-collaborative	4 (33%)
Healthcare providers involved	
Physician	3 (25%)
Nurse	4 (33%)
Other	0 (0%)
Type of EPOC intervention - At patient level:	
Education (e.g. lifestyle counselling)	10 (83%)
Reminder (e.g. drug adherence aids)	1 (8%)
- At healthcare provider level:	
Education (e.g. workshops)	4 (33%)
Feedback (e.g. medication change)	5 (42%)
Reminder (e.g. software tool)	0 (0%)
Number of interventions	
Mean (min, max)	3.8 (2, 5)
Duration of interventions, in months	
Mean (min, max)	5 (1, 9)
Frequency of interventions	
Once a month or more frequently	5 (42%)
Less than once a month	0 (0%)
Irregular or not clearly specified	7 (58%)
Duration of each intervention session, in minutes	
Mean (min, max)	30 (22.5, 37.5)

Figure S1 shows the results of the risk of bias assessment for each included study. Overall, the quality of the studies was relatively low. Six studies (50%) were at high risk of bias, six studies (50%) raised some concerns, and no study was classified as low risk. Out of the six studies with some concerns regarding risk of bias, four studies reported the mean difference in systolic BP.

### Effect of pharmacist interventions on blood pressure

**Fig. 2:**
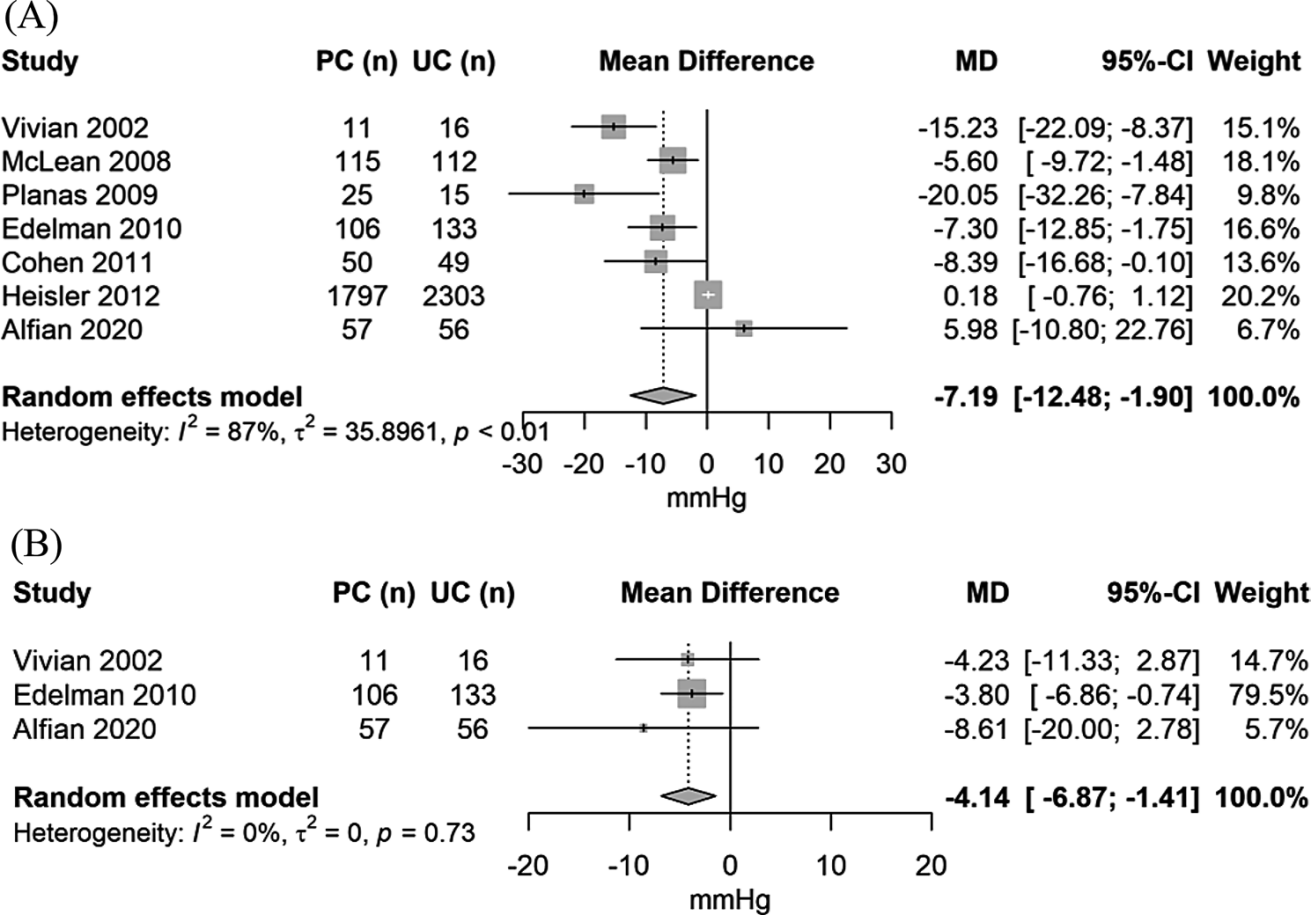
Forest plot of the mean difference between pharmacist intervention and usual care groups in (**A**) systolic and (**B**) diastolic blood pressure sorted by year of publication

The forest plots of the mean systolic (7 studies, *N* = 4,845) and diastolic (3 studies, *N* = 379) BP changes are displayed in Fig. 2. The pooled results show that pharmacist interventions conducted alone or in collaboration with other healthcare professionals reduced the mean systolic and diastolic BP by 7.2 mmHg (95% CI: -12.5 to -1.9; I^2^ = 87%) and 4.1 mmHg (95% CI: -6.9 to -1.4; I^2^ = 0%), respectively. There was a large between-study heterogeneity, with an I^2^ of 87% for systolic BP.

Sensitivity analysis excluding studies with high risk of bias resulted in a reduction by -4.4 mmHg systolic BP (95% CI: -8.7 to -0.1; I^2^ = 82%; 4 studies; *N* = 4,665 participants). The between-study heterogeneity remained high. The forest plot of the mean change in systolic BP restricted to studies with a relatively low risk of bias is displayed in Figure S2. Sensitivity analysis excluding studies of small size (less than 50 total participants) resulted in a mean difference between pharmacist interventions and usual care of -3.8 mmHg (95% CI: -7.9 to 0.3; I^2^ = 77%) for systolic BP (5 studies; *N* = 4,778 participants). These differences were of a slightly lower magnitude compared with the differences observed when all studies were included.

**Fig. 3:**
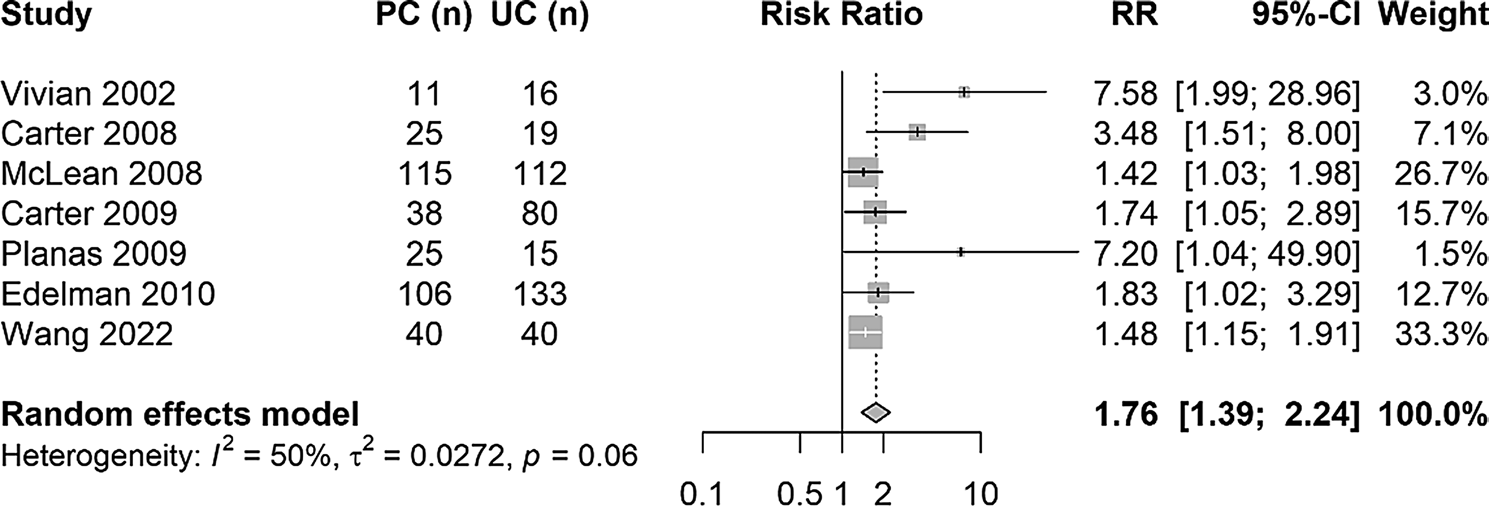
Forest plot of the relative risk (RR) between pharmacist intervention and usual care groups in blood pressure control sorted by year of publication

**Fig. 4:**
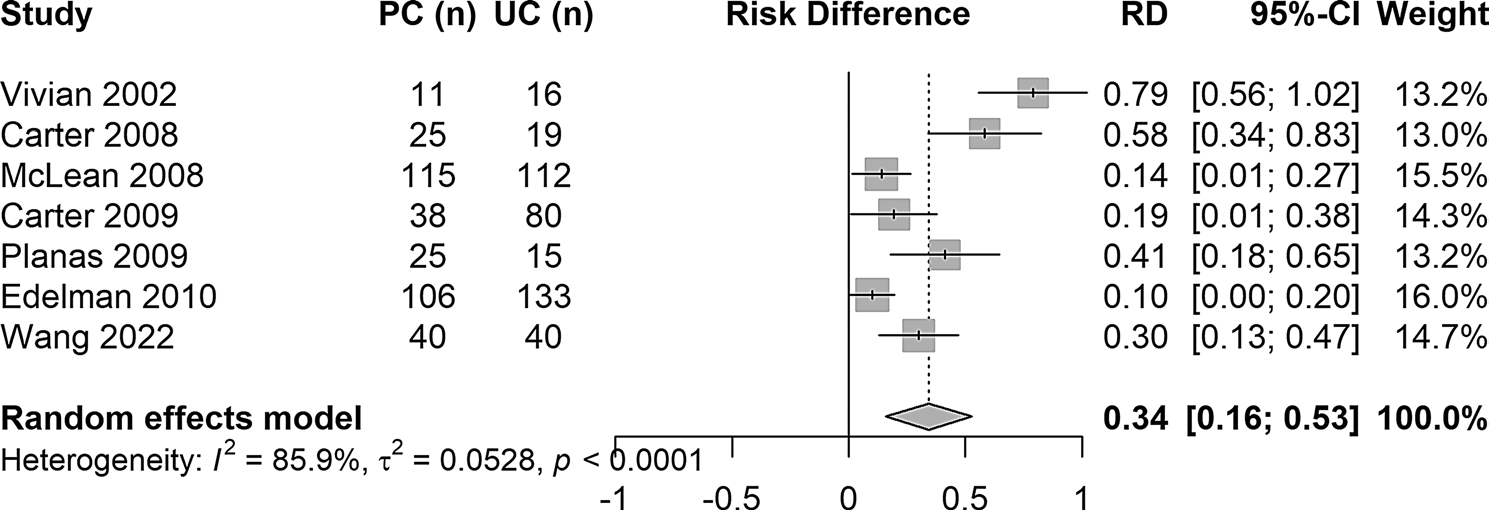
Forest plot of the risk difference (RD) between pharmacist intervention and usual care groups in blood pressure control change from baseline to follow-up sorted by year of publication

The secondary outcome, BP control at follow-up, was reported in 7 studies (*N* = 775 participants). At baseline, the median proportion of participants with controlled BP was 1.8% (25th to 75th percentile: 0.0%-15.9%) in the usual care group and 1.3% (25th to 75th percentile: 0.0%-12.7%) in the intervention group. At follow-up, the median proportion of participants with controlled BP was 23.5% (25th to 75th percentile: 12.0%-29.6%) in the usual care group and 48.0% (25th to 75th percentile: 46.3%-86.4%) in the intervention group. The pooled results showed a relative risk for BP control after pharmacist interventions of 1.76 (95% CI: 1.39 to 2.24; I^2^ = 50%; Fig. 3) compared to usual care. A subgroup analysis was conducted for BP control after pharmacist-led vs. interventions in collaboration (RR for BP control: pharmacist-led interventions 3.5, 95% CI: 1.0 to 11.7; in collaboration 1.7, 95% CI: 1.3 to 2.2; Figure S3), but the number of studies and participants, and therefore the statistical power, is limited. Pharmacist interventions increased BP control by + 34% points (95% CI: 16–53%; I^2^ = 85.9%; Fig. 4), measured in the pooled risk difference comparing the BP control change from baseline to follow-up between intervention and usual care groups.

### Reporting bias and GRADE assessment

Potential publication bias was observed for systolic BP as suggested by the asymmetry of the funnel plots (Figure S4), with Egger test p-value of 0.03 for systolic BP. However, due to the limited number of included studies, this analysis must be interpreted with caution. The GRADE framework was applied to assess the strength of the body of evidence for this systematic review (Table S4) [24]. The certainty of evidence for systolic BP was assessed as low when including all studies (the certainty was downgraded due to study limitations and publication bias).

## Discussion

Our systematic review and meta-analysis of 12 studies confirmed that pharmacist care resulted in a clinically and statistically significant reduction in BP in patients with diabetes and hypertension. The mean systolic reduction was − 7.2 mmHg (95% CI: -12.5 to -1.9; I^2^ = 87%) while the mean diastolic reduction was − 4.1 mmHg (95% CI: -6.9 to -1.4; I^2^ = 0%). There was also a large effect on BP control. A mean systolic BP reduction from baseline of ≥ 5 to 10 mm Hg is considered clinically meaningful as defined by the Hypertension Academic Research Consortium [40]. This is based on studies demonstrating that reductions “in office SBP of 5 and 10 mmHg are associated with ≈ 10% and ≈ 20% reductions in cardiovascular disease events, respectively, and independent of comorbidities such as cardiovascular disease” [40, 41]. Analyses restricted to relatively large or high-quality studies yielded slightly lower estimates. Our results showed that pharmacists’ interventions reduce BP, improve BP control, and support the involvement of pharmacists in BP management among patients with diabetes.

The BP reduction estimates found in our systematic review are comparable to estimates found in a previous systematic review (systolic BP: -6.2 mmHg, 95% CI: -4.6 to -7.8; diastolic BP: -4.5 mmHg, 95% CI: -2.8 to -6.2) [12]. This previous systematic review by Santschi et al. on the effect of pharmacist care on cardiovascular disease risk factors in patients with diabetes, synthesizing evidence up to the year 2012, applied less strict inclusion criteria (i.e., patients with diabetes, whether or not they had hypertension) resulting in a slightly higher number of studies (*n* = 15) [12]. We included randomized controlled trials, including patients with diagnoses of both hypertension and diabetes, with a recent search in 2024 while adhering to established guidelines, such as the Cochrane guidelines for Systematic Reviews and the PRISMA statement [13, 15]. Sensitivity analysis excluding studies with high risk of bias led to estimates of a slightly lower magnitude compared with the differences observed when all studies were included. Very few studies also reported the effect of interventions on diabetes-related outcomes. Four included studies report results on HbA1c change or HbA1c level at follow-up [29–31, 36]. All four studies reported larger HbA1c reduction after pharmacist interventions compared to the control groups (between − 0.2% and − 1.2%).

When interpreting the results of this study, several limitations should be considered. First, trials for such interventions face practical challenges in blinding both participants and researchers, increasing the risk of bias. Hence, we found no study at low risk of bias, and many included studies were considered at high risk. This limitation also affected the GRADE assessment of the certainty of the evidence, resulting in a low-certainty rating. However, a recent review concluded that it is rare to fulfil the criteria for high-quality evidence with GRADE [42]. Second, our analyses indicated potential publication biases, i.e., a preference for publishing studies with favourable results over those reporting null or negative findings. Thus, our estimates might be biased toward a greater effect of pharmacist interventions. Third, another limitation is that we could not assess if the effect of pharmacist interventions differs depending on age and sex of the participants, which would require individual participant data (IPD) meta-analysis. The limited numbers of studies included in this review also restrains the possibility of such sub-group analyses. We did not run analyses by region. However, in our previous systematic review of pharmacist care in hypertension management, which was not limited to patients with diabetes, a larger number of studies (*n* = 95) were included, and we did not find differences in the effect of pharmacist care on BP control by geographical region [43]. Fourth, we could not estimate if the effect of intervention differed if patients were treated or not at baseline, and to which extend the effect was due to treatment initiation or intensification. Such analysis would also require IPD analyses. However, in several studies, there were evidence that treatment intensification was the cause of BP improvement.

Finally, a key limitation, is the high heterogeneity observed in the included studies. This high heterogeneity may be explained by the large variation in interventions and settings (Tables 1 and 2, Table S3). The included studies also had different patient populations, e.g., some included only patients with uncontrolled hypertension, other with a mix of patients with controlled and uncontrolled hypertension at baseline (Table S3). One major issue with such a review with meta-analysis is that interventions are complex and differ from one study to the other. They can have multiple layers, involve different healthcare providers, and be of different intensity. As a result, pooling the results across studies is disputable and give merely an idea of the expected effect on BP. There is no simple way to overcome this limitation. We have described in detail each study in the appendix to document this heterogeneity (see Supplementary Table S3). However, it calls for further analyses to identify what work best, e.g. through network meta-analysis and IPD analysis. Additional limitations include the absence of specific information regarding usual care in numerous studies and the relatively low number of studies with a long follow-up. Therefore, it is difficult to know what exactly the pharmacists’ interventions on blood pressure are compared to and their long-term impact. Of note, we identified one study conducted by Carter et al. in which the intervention consisted of healthcare provider education notably by training them to overcome clinical inertia and to strengthening communication between physicians and pharmacists [26]. While the effect on BP control was substantial, further research is needed to confirm the effectiveness of this type of intervention.

The American Diabetes Association’s “Standards of Care in Diabetes” recommends “incorporating care management teams including nurses, dietitians, pharmacists, and other health care professionals” [44]. The 2016 European Guidelines on Cardiovascular Disease Prevention (jointly by 10 professional societies, including the European Society of Cardiology and the European Association for the Study of Diabetes) recommend to “introduce physician assistants and/or trained nurses or pharmacists whenever it is necessary and feasible” and acknowledge that “collaboration with pharmacists or pharmacist-directed care was superior to standard care with respect to BP, total cholesterol and LDL-C levels” [45]. The Task Force on diabetes, pre-diabetes, and cardiovascular diseases of the European Society of Cardiology developed in collaboration with the European Association for the Study of Diabetes writes that “multifaceted strategies are most effectively delivered through multidisciplinary teams”, but does not refer to pharmacists specifically [46]. Further attention to the intersection of diabetes and hypertension management, including pharmacist care, and more details on effective team-based care interventions are needed in clinical practice guidelines. The regional differences in health care have been recognized with the 2016 European Guidelines on Cardiovascular Disease Prevention recommending that “patient follow-up should be carried out by the health care team, which should include physicians, nurses and pharmacists in a concerted activity, although wide variations exist in the organization of health care systems across Europe” [45]. We did not identify any study conducted in Europe, hence the need for more evidence for pharmacist and team-based care interventions in Europe or outside North America.

In conclusion, our systematic review provides consistent evidence in support of pharmacist interventions in managing hypertension among patients with diabetes. The heterogeneity of observed effects in our analyses suggests that intervention program should include rigorous impact evaluation and monitoring of patient-reported outcomes and implementation processes, as well as cost-effectiveness analyses.

## Supplementary Information

Below is the link to the electronic supplementary material.

Supplementary Material 1

## Data Availability

Data for this review were retrieved from published papers. No additional data are available.
