# Supplementary material for: Pharmacist interventions to improve hypertension management among patients with diabetes: a systematic review and meta-analysis of randomized controlled trials
Source: BMC Health Serv Res. 2025 Oct 1;25:1268. doi: 10.1186/s12913-025-13461-7 (PMC12487230; doi:10.1186/s12913-025-13461-7)
Supplement: Supplementary file 1 — Supplementary Material 1 [file 12913_2025_13461_MOESM1_ESM.docx]

**SUPPLEMENTAL MATERIAL**

Pharmacist interventions to improve hypertension management among patients with diabetes: a systematic review and meta-analysis of randomized controlled trials

Viktoria Gastens, Stefano Tancredi, Dylan Bonnan, Blanche Kiszio, Cinzia Del Giovane, Ross T. Tsuyuki, Gilles Paradis, Arnaud Chiolero, Line Guénette, Valérie Santschi

**Supplemental Table S1.** Full search strategies for all databases, registers, and websites, including any filters and limits used.

**Supplemental Table S2.** Independently extracted data during the systematic review process.

**Supplemental Table S3.** Study and pharmacist intervention characteristics for each included study.

**Supplemental Table S4.** Grading of Recommendations Assessment, Development and Evaluation (GRADE) assessment of the evidence for the effect of pharmacist interventions on systolic blood pressure (BP).

**Supplemental Figure S1.** Results of the risk of bias assessment using the revised Cochrane risk of bias (RoB 2) tool and visualised with the robvis tool.

**Supplemental Figure S2.** Sensitivity analysis limited to relatively high-quality studies. Forest plot of the mean difference between pharmacist and usual care group in systolic blood pressure sorted by year of publication.

**Supplemental Figure S3.** Subgroup analysis and forest plot of the relative risk between pharmacist and usual care group in blood pressure control sorted by year of publication comparing pharmacist-directed against pharmacist-collaborative care.

**Supplemental Figure S4.** Funnel plot to assess publication bias for systolic blood pressure.

**Supplemental Table S1.** Full search strategies for all databases, registers, and websites, including any filters and limits used.

| Ovid Medline Search Strategy | 1. Pharmacists/ or Community Pharmacy Services/ or Pharmaceutical Services/ or Pharmaceutical Services, Online/ or Pharmacy Service, Hospital/ or Pharmacies/ or Pharmacy/ or Evidence-Based Pharmacy Practice/ or Pharmacy research/ or Drug Information Services/ or Medication Therapy Management/ or Patient Care Team/ or ("pharmacist*" or "pharmaceutical intervention*" or "pharmaceutical care" or "pharmacies" or "pharmacist-led" or "team-based care").tw.  2. Hypertension/ or essential hypertension/ or hypertension, malignant/ or Antihypertensive Agents/ or [Blood Pressure](https://ovidsp.dc2.ovid.com/ovid-b/ovidweb.cgi?&S=NBMBFPELEGEBDCBLIPPJOEOGHANJAA00&Search+Link=%22Blood+Pressure%22%2f)/ or Blood Pressure Monitoring, Ambulatory/ or (hypertension or "high blood pressure" or "blood pressure management" or "blood pressure control" or "blood pressure monitoring" or "blood pressure telemonitoring" or "changes of blood pressure" or "hypertensive disease*" or "antihypertensive" or "antihypertensive agents" or "high bp" or "bp raised" or "bp control").tw. not ("pulmonary hypertension".tw.)  3. ((randomized controlled trial or controlled clinical trial).pt. or randomized.ab. or placebo.ab. or drug therapy.fs. or randomly.ab. or trial.ab. or groups.ab.) not ("trial registration number".tw.)  4. 1 AND 2 AND 3 |
| --- | --- |
| Embase Search Strategy | 1. 'pharmacist'/exp OR 'clinical pharmacy'/de OR 'evidence-based pharmacy'/de OR 'pharmacy research'/de OR 'medication therapy management'/de OR 'pharmaceutical care'/de OR 'pharmacist attitude'/de  2. 'hypertension'/de OR 'diabetic hypertension'/de OR 'essential hypertension'/de OR 'hereditary hypertension'/de OR 'hypertensive crisis'/de OR 'malignant hypertension'/de OR 'orthostatic hypertension'/de OR 'resistant hypertension'/de OR 'antihypertensive agent'/de OR 'elevated blood pressure'/de OR 'blood pressure monitoring'/de OR 'blood pressure measurement'/exp OR 'blood pressure regulation'/exp OR 'blood pressure fluctuation'/exp OR 'antihypertensive therapy'/de OR 'antihypertensive activity'/de OR 'hypertensive patient'/de  3. 'crossover procedure':de OR 'double-blind procedure':de OR 'randomized controlled trial':de OR 'single-blind procedure':de OR (random* OR factorial* OR crossover* OR cross NEXT/1 over* OR placebo* OR doubl* NEAR/1 blind* OR singl* NEAR/1 blind* OR assign* OR allocat* OR volunteer*):de,ab,ti NOT 'Conference Abstract'  4. NOT [medline]/lim  5. 1 and 2 and 3 not 4 |
| Cochrane Central Search Strategy | 1. Pharmacists or "Community Pharmacy Services" or "Pharmaceutical Services" or "Pharmaceutical Services, Online" or "Pharmacy Service, Hospital" or "Pharmacies" or "Pharmacy" or "Evidence-Based Pharmacy Practice" or "Pharmacy research" or "Drug Information Services" or "Medication Therapy Management" or "Patient Care Team" in Title Abstract Keyword  2. Hypertension or "essential hypertension" or "hypertension, malignant" or "Antihypertensive Agents" or "Blood Pressure Monitoring, Ambulatory" in Title Abstract Keyword - (Word variations have been searched)  3. 1 AND 2 |
| JBI Search Strategy | 1. Pharmacist* or "Community Pharmacy" or "Pharmaceutical Services" or "Pharmacy Service" or Pharmacies or Pharmacy or "Evidence-Based Pharmacy Practice" or "Drug Information Services" or "Medication Therapy Management" or "Patient Care Team" or "Pharmaceutical intervention*" or "Pharmaceutical care" or "pharmacist-led" or "team-based care"  2. Hypertension or "Antihypertensive Agents" or "Blood Pressure Monitoring" or "high blood pressure" or "blood pressure management" or "blood pressure control" or "blood pressure telemonitoring" or "changes of blood pressure" or "hypertensive disease*" or "antihypertensive"  3. 1 AND 2 |
| CINAHL Search Strategy | 1. (MH "Pharmacists") OR (MH "Pharmacist Attitudes") OR (MH "Pharmacy Service") OR (MH "Medication Management") OR (MH "Pharmacy, Retail") OR (MH "Drug Information Services") OR (MH "Prescription Drug Monitoring Programs") OR (MH "Medication Management") OR (MH "Multidisciplinary Care Team")  2. (MH "Hypertension") OR (MH "Essential Hypertension") OR (MH "Hypertension, Malignant") OR (MH "Hypertension, Isolated Systolic") OR (MH "Hypertensive Crisis") OR (MH "Antihypertensive Agents") OR OR (MH "Blood Pressure Determination") OR (MH "Blood Pressure Monitoring, Ambulatory")  3. (randomized controlled trials OR MH double-blind studies OR MH single-blind studies OR MH random assignment OR MH pretest-posttest design OR MH cluster sample OR TI (randomised OR randomized) OR AB (random*) OR TI (trial) OR (MH (sample size) AND AB (assigned OR allocated OR control)) OR MH (placebos) OR PT (randomized controlled trial) OR AB (control W5 group) OR MH (crossover design) OR MH (comparative studies) OR AB (cluster W3 RCT)) NOT ((MH animals+ OR MH animal studies OR TI animal model*) NOT MH human)  4. 1 AND 2 AND 3 |
| Web of science Search Strategy | 1. Pharmacist* or “Community Pharmacy” or “Pharmaceutical Services” or “Pharmacy Service” or Pharmacies or Pharmacy or “Evidence-Based Pharmacy Practice” or “Drug Information Services” or “Medication Therapy Management” or “Patient Care Team” or "Pharmaceutical intervention*" or "Pharmaceutical care" or "pharmacist-led" or "team-based care"  2. Hypertension or “Antihypertensive Agents” or “Blood Pressure Monitoring” or "high blood pressure" or "blood pressure management" or "blood pressure control" or "blood pressure telemonitoring" or "changes of blood pressure" or "hypertensive disease*" or "antihypertensive"  3. “randomized controlled trial*” OR “double-blind studie*” OR “single-blind studie*” OR “random assignment” OR “pretest-posttest design” OR “cluster sample”  4. 1 AND 2 AND 3 |
| Tripdatabase Search Strategy | pharmacist intervention hypertension  Filter: rct |
| Grey literature Search Strategy | (pharmacist OR pharmacists) AND (hypertension OR antihypertensive OR "high blood pressure") |

**Supplemental Table S2.** Independently extracted data during the systematic review process.

| **Type of data** | **Items** |
| --- | --- |
| 1. Study identification | Author(s), year of publication, study country. |
| 2. Study characteristics | Setting and design.  Study duration, frequency of follow-up.  Randomization, blinding.  Sample size (total and per arm). |
| 3. Participants characteristics | Number of participants allocated to each group, number of patients analyzed.  Mean age, age range, sex.  Diabetes, other comorbidities, cardiovascular risk factors (smoking, dyslipidaemia).  Drug intake. |
| 4. Usual care (control group) characteristics | Healthcare providers involved.  Frequency of follow-up. |
| 5. Intervention characteristics | Type of interventions (pharmacist directed or in collaboration).  Duration of intervention.  Description of interventions: key components, frequency, format (noting if the detail provided is enough for replication), healthcare providers involved.  Cochrane Effective Practice and Organization of Care (EPOC) taxonomy. |
| 6. Outcomes for each group | Mean BP change and standard error (or confidence interval, p value) between baseline and follow-up.  Mean BP and SD at baseline and follow-up.  BP control (% reaching a pre-defined BP target) at baseline and follow-up.  Method of BP measurement.  Mean HbA1c change and standard error (or confidence interval, p value) between baseline and follow-up.  Mean HbA1c and SD at baseline and follow-up.  HbA1c control (% reaching a pre-defined target) at baseline and follow-up.  Glucose (mg/dl) at baseline and follow-up. |

**Supplemental Table S3.** Study and pharmacist intervention characteristics for each included study.

| **Source; year of publication and country** | **Study setting** | **Study design, duration** | **Sample size (intervention/usual care) in analysis** | **Participants; mean age** | **Key components of pharmacist interventions** | **EPOC category (classified by VG, ST)** | **Intervention frequency, duration** | **Description of usual care group** | **Outcomes** |
| --- | --- | --- | --- | --- | --- | --- | --- | --- | --- |
| ***Pharmacist directed care*** | | | | | | | | | |
| Vivian et al; [1] 2002 United States | Outpatient clinic | RCT, 6 months | 53 (26/27), 27 (11/16) for subgroup with diabetes | Uncontrolled hypertensive patients (BP≥140/90 mmHg) taking antihypertensive Med; 65 y | Counseling of Med and lifestyle; Assessment of compliance; Change in HT Med (drug selection and dosage) | Patient education  Healthcare provider feedback | Monthly | Patients in the control group received traditional pharmacy services (dispensing, brief counseling about drugs, and review of drug profiles) but did not make monthly visits to the pharmacist-managed hypertension clinic. They received care from their primary care providers as needed, at least once/year. All primary care providers were informed that their patients were enrolled in the study | SBP after end of study in the intervention group group=132.2 mmHg and in the control 147.43 mmHg (p=0.0002). (Table 3)  DBP after end of study in the intervention group group=74.2 mmHg and in the control 78.43 mmHg (p=0.254). (Table 3)  Of the 11 patients with diabetes in the intervention group, 10 (91%) attained the goal blood pressure of below 130/80 mm Hg versus only two (12 %) of 16 patients  with diabetes in the control group (p=0.001;  Table 3). |
| Planas et al; [2] 2009 United States | Community pharmacy | RCT, 9 months | 40 (25/15) | Patients with uncontrolled DM (HbA1c>7.0%) and HT (BP≥130/80mmHg) or taking antihypertensive Med; 65 y | A community-based Med therapy management program:  1. Med review related to current prescribed and non-prescribed Med to identify DRPs;  2. DRPs identification;  3. If DRP identified, recommendations to physicians regarding adjustment HTA Med dose and addition Med by fax or telephone;  4. Patient education regarding Med, lifestyle and diet;  5. A copy of the visit note sent to physician | Patient education  Healthcare provider feedback | Monthly | Participants in the control group attended visits at baseline and 3, 6, and 9 months, during which their BP was recorded, and they were informed of BP goals for patients with diabetes | Difference in SBP change between  the control and intervention groups= 20.05 mmHg (95%CI= 7.45 - 32.66) |
| Heisler et al; [3] 2012 United States | Outpatient clinic | Cluster-RCT, 26 months | 4100 (1797/2303) | Patients with DM and HT with poor BP control; 65 y | Pharmacist training (motivational interviewing training), medication changes, patient counseling (BP self-monitoring, adherence barriers, discussed lab values), BP measurement | Healthcare provider education  Patient education | Scheduled visits over 14 months until discharge (all medication adherence issues had been addressed; home or clinic BPs were at target; or the patient was on maximum tolerated medications) | Standard healthcare services through their primary care provider, which in all sites included access to care manager and other control clinical pharmacist services targeting patients with diabetes mellitus with poor risk factor control. The study team had no contact with the usual care teams, nor did the intervention clinical pharmacists who worked exclusively with intervention team patients | In the primary analysis, the intervention group SBP change from the 6 months before versus 6 months after the 14-month intervention was not different from control group, declining 8.9 mm Hg in the intervention group in comparison with a 9.0 mm Hg decrease for the control group (difference of 0.18 [0.77, 1.13])  There were no differences in mean A1c between intervention and control teams after the end of the intervention period (examining  a 12-month period): A1c mean of 7.4% (1.4) and 7.6%  (1.6) on control teams. |
| Alfian et al; [4] 2020 Indonesia | Outpatient Clinic | RCT, 3 months | 113 (56/57) | Patients with DM2 and HT aged at least 18 years old, diagnosed with DM2 for at least 1 year, using at least one antihypertensive drug in the last 3 months and non-adherent to the HT treatment; 63 y* | Discussed patient-specific barriers for medication adherence. Simple question-based flowcharts and adherence intervention wheel provided to pharmacy staff. Goal setting. | Patient education | 2 visits (baseline, 1 months) | Received pharmacist counselling based on the Indonesian guideline of pharmacy practice | Mean difference in SBP=5.98 mmHg, 95%CI= -10.8, 22.76.  Mean difference in DBP= -8.61 mmHg, 95%CI= -20.01, 2.78 |
| Contreras-Vergara et al; [5] 2022 Mexico | Outpatient clinic | RCT, 6 months | 89 (46/43) | Patients between 18 and 60 years old with HT and DM; 56 y | Patient education (disease, lifestyle, medication, orally and written, wallet card) | Patient education | 3 visits (baseline, 3, 6 months), for 20-25 minutes | Control group received regular (standard) education from their physician at each of their office visit | SBP at the end of study period 141.14 mmHg in control group and 130.15 mmHg in the intervention group.  DBP at the end of study period 92.91 mmHg in control group and 87 mmHg in the intervention group.  HbA1c at the end of study period 8.8 % in control group and 7.6 % in intervention group. |
| Malik et al; [6] 2022 Pakistan | Community pharmacy | Cluster RCT, 6 months | 80 (40/40) | Patients diagnosed with DM (Type I or II) and HT with an HbA1c value ≥ 7% and BP greater than 140/90 mmHg at the time of diagnosis; 44 y* | Training aids for pharmacists (diabetes, hypertension, log sheets, glucometer, BP measuring devices, questionnaires), patient kits (disease brochures, diet charts, BP and glucose monitoring cards), oral patient counselling, BP and blood glucose measurement | Healthcare provider education  Patient education | Every 15 days, for 6 months, minimum 20 minutes | Control group received the usual pharmacy services, i.e., dispensing medications and providing information regarding medication administration. Patients enrolled in the control and intervention group were required to visit the community pharmacy every 15 days for 6 months during the study | SBP at 6 months=145.48 mmHg in control group and 130.10 mmHg in the intervention group.  DBP at 6 months=97.00 mmHg in control group and 88.83 mmHg in the intervention group |
| Wang et al; [7] 2022 China | Outpatient clinic | RCT, 3 months | 80 (40/40) | Patients with DM2 and HT, aged between 18 and 65 years, admitted to the hospital for failing to control the blood glucose or BP, DM2and HT were well controlled according to the evaluation of physicians at discharge, received pharmaceutical care during hospitalization, and receiving antidiabetic and antihypertensive drugs after discharge; 42 y | 1. Consultations by clinical pharmacists after the visit with the physician.  2. Design of standardized science education materials.  3. Establishment of a “follow-up service,” WeChat exchange group and WeChat official account.  4. Continuous individualized follow-up. After discharge, the participants were followed by telephone every two weeks. | Patient education  Patient reminder | Over 3 months | Participants returned to the hospital every 2 weeks for regular clinical follow-up, including the prescription of hypoglycemic and antihypertensive drugs and the evaluation of blood sugar, BP, and other indexes. Nursing staff followed the participants by telephone once a month and recorded the participants’ blood sugar, BP, and medication compliance | BP control rate at 3 months 62.5% in routine group and 92.5% in pharmaceutical care group (p<0.001).  HbA1c median at 3 months 6.95% in routine group and 6.45% in pharmaceutical care group (p=0.007). (Table 2) |
| ***Pharmacist collaborative care*** | | | | | | | | | |
| Carter et al; [8] 2008 United States | Outpatient clinic | cluster- RCT, 9 months | 179 (101/78), 44 (25/19) for subgroup with diabetes | Uncontrolled hypertensive patients (BP 145- 179/95-109 mmHg; BP 135-179/85-109 mmHg if DM) taking antihypertensive Med or not; 61 y | Patient interview related to Med; Verbally or written recommendation to physician regarding HT Med changes; Recommendation of Med compliance aids if necessary.  Team members: physician and nurse | Healthcare provider education | Pharmacists were encouraged to attend each clinic visit (2, 4, 6, and 8 months), and they were encouraged to initiate additional visits or telephone contact if BP remained uncontrolled | Patients in both groups were given written information on hypertension. The research nurses encouraged all patients to follow the lifestyle modifications. Patients were also made aware of their goal BP level | At 9 months, for patients with diabetes, BP was controlled at <130/80 mm Hg in 23.5% of patients in  the control group and 81.8% in the intervention  group (adjusted odds ratio, 40.1; CI, 4.1–394.7;  P=.002). |
| McLean et al; [9] 2008 Canada | Community pharmacy | RCT, 6 months | 227 (115/112) | Adult patients with DM and with BP>130/80 mmHg taking insulin or oral hypoglycemic Med for >6 months; 65 y | Pharmacist-nurse team including:  1. Patient education and counseling regarding cardiovascular risk reduction;  2. Distribution of HT education pamphlet and wallet card documenting recorded patient BP measures faxed to physicians;  3. Patient's risk factors, current Med and BP measures with any suggestions for further management based on guidelines faxed to physicians.  Team members: nurse | Patient education  Healthcare provider feedback | 6 weeks intervals, over 24 weeks | Patients randomized to usual care received the same BP wallet card with their BP measures documented, a pamphlet on diabetes, and general diabetes counseling from the nurse or pharmacist. Usual care patients received telephone follow-up at 12 weeks and no other follow-up until the in-person close-out visit at 24 weeks. Neither of these visits entailed any therapeutic advice to the usual care patients and were merely for the collection of end point data | The mean between-group difference in SBP was 5.6 (SE=2.1) mmHg (p=0.008) |
| Carter et al; [10] 2009 United States | Outpatient clinic | Cluster- RCT, 6 months | 402 (192/210), 118 (38/80) for subgroup with diabetes | Uncontrolled hypertensive patients (BP 140-179/90-109 mmHg; BP 130-179/80-109 mmHg if DM) taking 0 to 3 antihypertensive Med; 58 y | Assessment and adjustment of HT med approved by physicians; assessment of BP; verbally drug recommendations to physicians; education to physician if necessary.  Team members: physician | Healthcare provider education  Healthcare provider feedback | Pharmacists were encouraged to assess medications and BP at baseline and at 1 month and by telephone at 3 months and more frequently if necessary | Clinical pharmacists at control sites abstained from providing care for study patients but continued to answer general treatment questions from physicians | Blood pressure was controlled in 26.1% of patients with  diabetes mellitus in the control group and in 45.5% of patients with diabetes mellitus in the intervention group  (adjusted odds ratio, 4.7; 95% CI, 1.7-13.1; P=.003). |
| Edelman et al; [11] 2010 United States | Outpatient clinic | RCT, 12  months | 239 (133/106) | Patients with uncontrolled DM (HbA1c>7.5%) and HT (BP>140/90 mmHg) taking Med for DM; 62 y | At each group medical clinics session (comprising 7 to 8 patients with the care team):  1. Med review from medical records by pharmacist and physician;  2. Reviewing of BP and home blood glucose readings by pharmacist and physician;  3. Development of individualized plan for Med or lifestyle management with pharmacist and physician;  4. Adjustment of Med by pharmacist and physician and report to primary care providers; 5. Patient education and counseling related to Med and lifestyle.  Team members: physician and nurse | Patient education  Healthcare provider feedback | The groups met every 2 months (7 visits over 12 months). Sessions lasted 90-120 minutes | Patients in the usual care group received no active intervention | Mean difference (95% CI) between groups in SBP=-7.3 mmHg (-12.8 to -1.7). (Table 2)  Mean difference (95% CI) between groups in DBP=-3.8 mmHg (-6.9 to -0.8). (Table 2)  Mean difference (95% CI) between groups in final HbA1c level=-0.33 (-0.80 to 0.13). (Table 2) |
| Cohen et al; [12] 2011 United States | Outpatient Clinic | RCT, 6 months | 99 (50/49) | Patients with a diagnosis of DM2; HbA1c > 7.0%, LDL > 100 mg/dL (2.59 mmol/L) or LDL > 70 mg/dL (1.81 mmol/L) for those with coronary artery disease, and BP > 130/80 mm Hg, each documented at least once in the medical records in the 6 months before enrollment; 69 y | Education; behavioral and pharmacologic interventions for hypertension, hyperlipidemia, and hyperglycemia and tobacco use  Team members: nurses, dietitians, physical therapists | Patient education | 4 once-weekly 2-hour sessions, followed by 5 monthly booster session. The booster sessions lasted 90 minutes. | Standard of care. visits once every 4 months on average. The primary care providers have access to the same electronic medical record, which contains clinical reminders, computer-based references, drug formulary information, and referral services to diabetes self-management education, nutrition, physical therapy, and the weight loss program MOVE! | SBP at the end of study period=−9.19 (−14.95 to −3.43) in the intervention group and −0.80 (−5.61 to 4.02) in the control group |

Abbreviations: RCT: randomized controlled trial; HT: hypertension; Med: medication; BP: blood pressure; SBP: systolic blood pressure; DBP: diastolic blood pressure; ITT: intention-to-treat analyses; SE: standard error; GP: general practitioner; DRP: drug related problems; HbA1c: glycated haemoglobin; DM: diabetes mellitus; DM2: diabetes mellitus type 2; AOBP: automated office blood pressure; ABPM: ambulatory blood pression monitoring; CKD: chronic kidney disease CVD: cardiovascular; COPD: chronic obstructive pulmonary disease; LDL: low-density lipoproteins

*: The mean age was not reported and was computed using the age categories

**: age was not reported

**Supplemental Table S4.** Grading of Recommendations Assessment, Development and Evaluation (GRADE) assessment of the evidence for the effect of pharmacist interventions on systolic blood pressure (BP).

|  | **Outcome** | **Mean difference (95% CI) with pharmacist interventions** | **N participants (N studies)** | **Certainty (GRADE assessment)** |
| --- | --- | --- | --- | --- |
| All studies | Systolic BP | 7.2 mmHg lower (between 1.9 and 12.5 mmHg) | 4 845 (7 RCTs) | Low^a^ |

CI= confidence interval

^a^ downgraded to “Low” due to several studies at high risk of bias and possible publication bias

**Supplemental Figure S1.** Results of the risk of bias assessment using the revised Cochrane risk of bias (RoB 2) tool and visualised with the robvis tool. [13, 14]


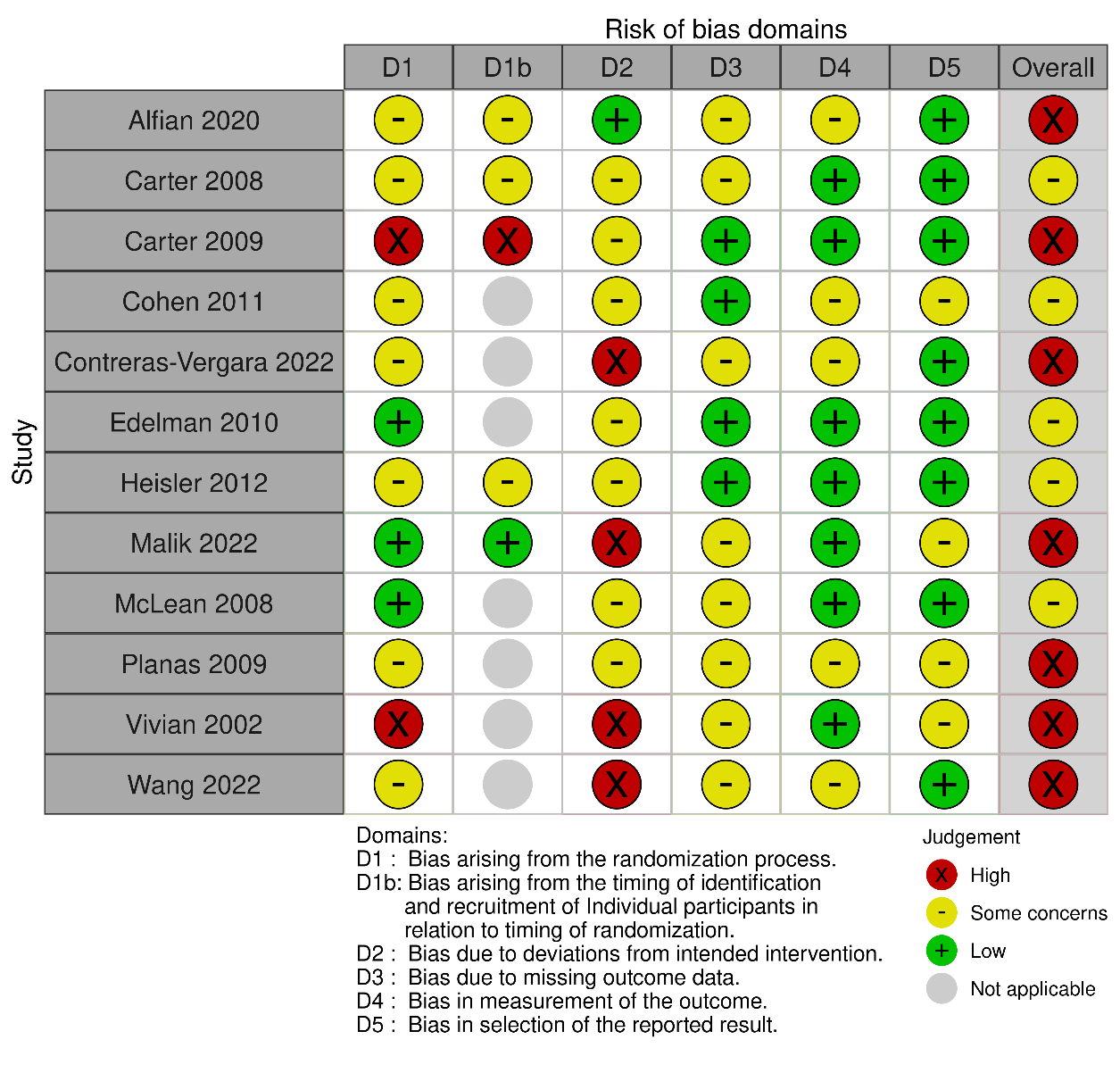


**Supplemental Figure S2.** Sensitivity analysis limited to relatively high-quality studies. Forest plot of the mean difference between pharmacist and usual care group in systolic blood pressure sorted by year of publication.


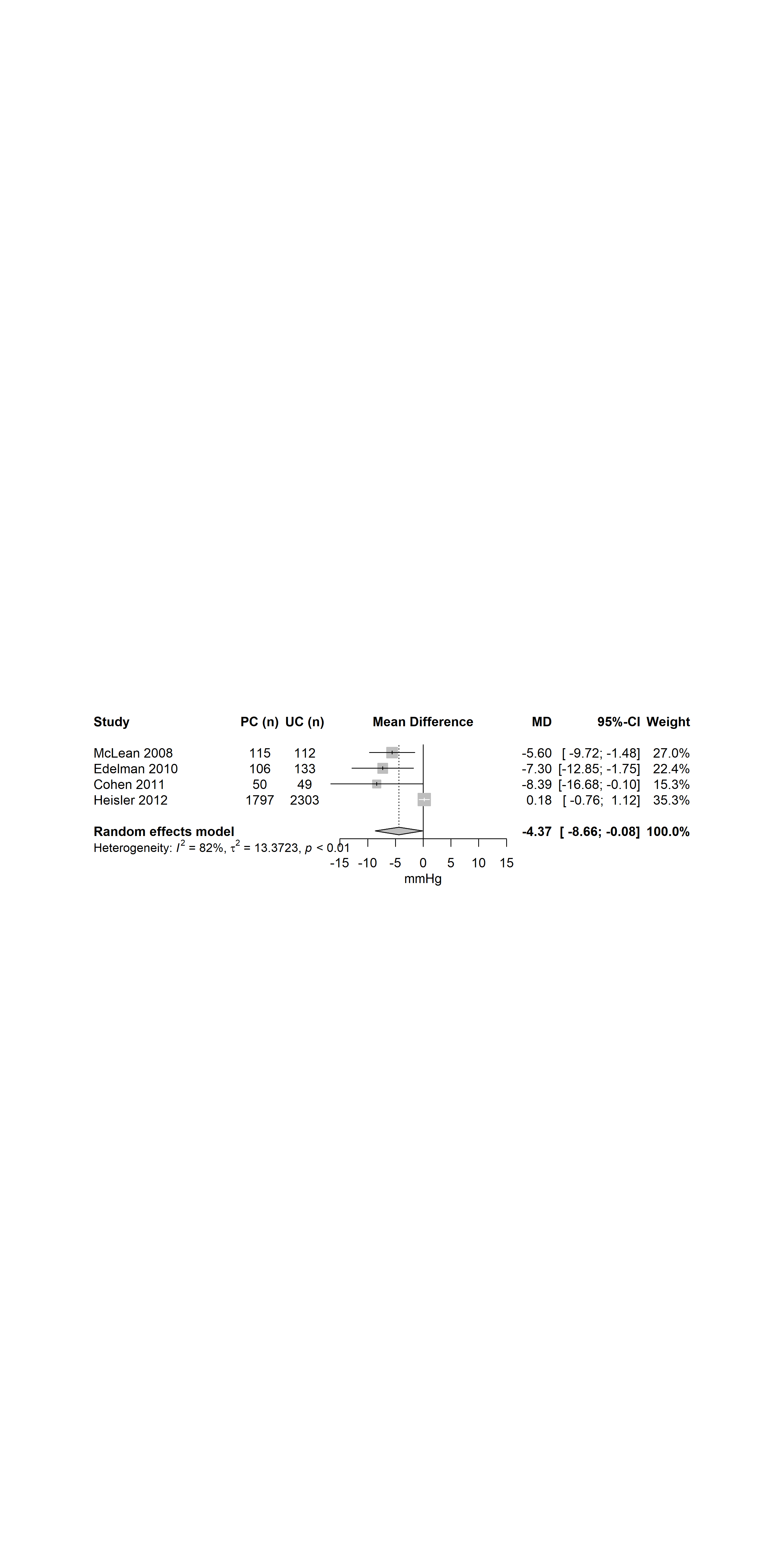


**Supplemental Figure S3.** Subgroup analysis and forest plot of the relative risk between pharmacist and usual care group in blood pressure control sorted by year of publication comparing pharmacist-directed against pharmacist-collaborative care.

**
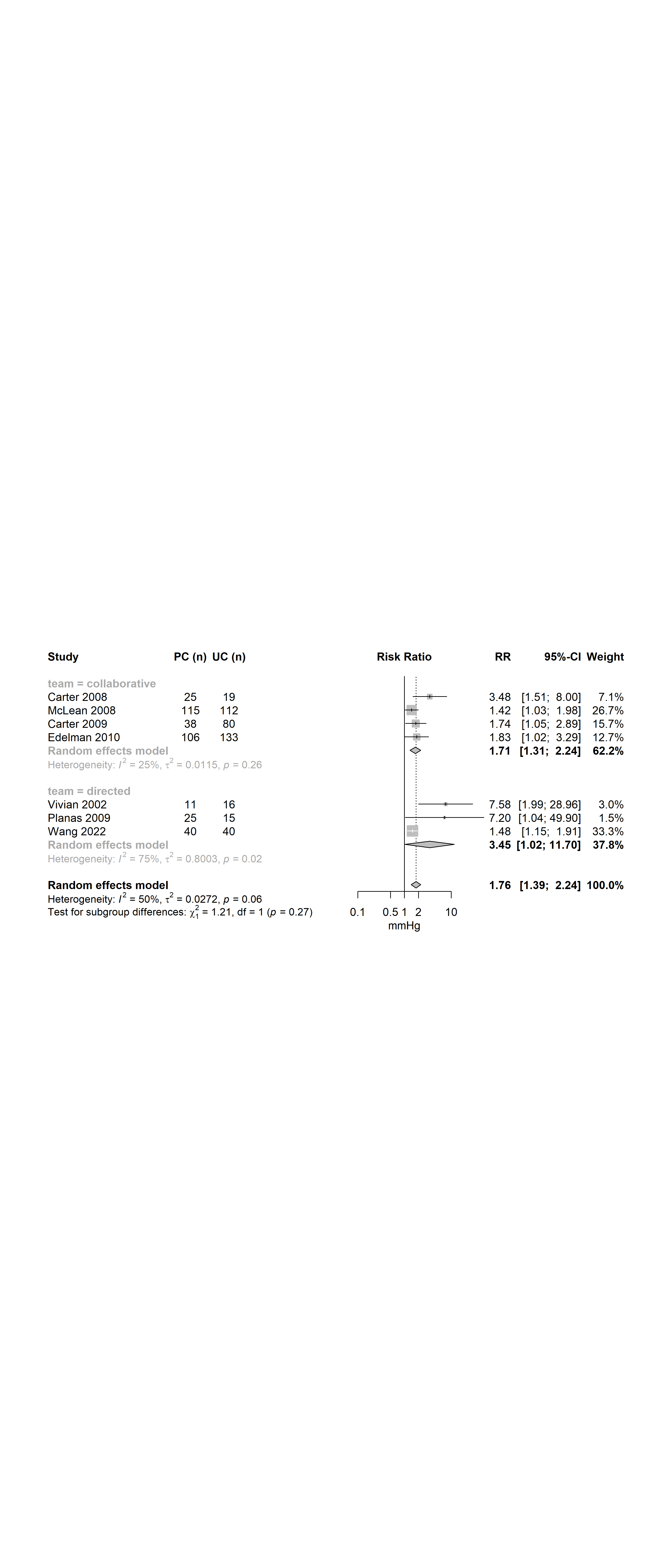
**

**Supplemental Figure S4.** Funnel plot to assess publication bias for the primary outcome systolic BP change.


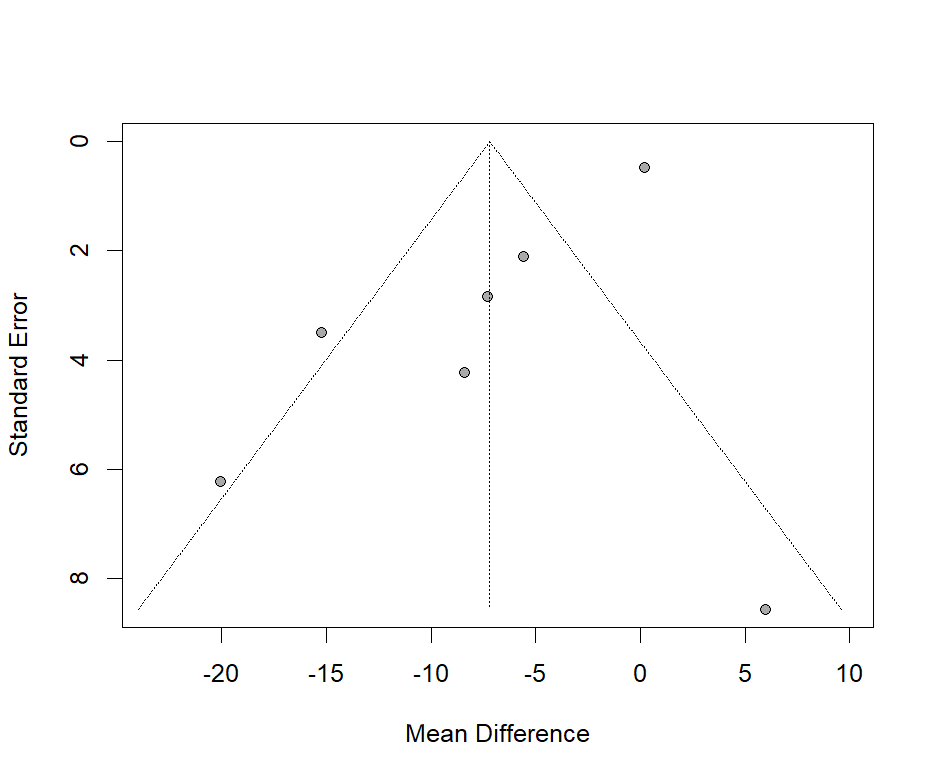


**REFERENCES**

| [1] | E. M. Vivian, "Improving blood pressure control in a pharmacist-managed hypertension clinic," *Pharmacotherapy: The Journal of Human Pharmacology and Drug Therapy,* vol. 22, p. 1533–1540, 2002. |
| --- | --- |
| [2] | L. G. Planas, K. M. Crosby, K. D. Mitchell and K. C. Farmer, "Evaluation of a hypertension medication therapy management program in patients with diabetes," *Journal of the American Pharmacists Association,* vol. 49, p. 164–170, 2009. |
| [3] | M. Heisler, T. P. Hofer, J. A. Schmittdiel, J. V. Selby, M. L. Klamerus, H. B. Bosworth, M. Bermann and E. A. Kerr, "Improving blood pressure control through a clinical pharmacist outreach program in patients with diabetes mellitus in 2 high-performing health systems: the adherence and intensification of medications cluster randomized, controlled pragmatic trial," *Circulation,* vol. 125, p. 2863–2872, 2012. |
| [4] | S. D. Alfian, J. F. M. van Boven, R. Abdulah, H. Sukandar, P. Denig and E. Hak, "Effectiveness of a targeted and tailored pharmacist-led intervention to improve adherence to antihypertensive drugs among patients with type 2 diabetes in Indonesia: A cluster randomised controlled trial," *British journal of clinical pharmacology,* vol. 87, p. 2032–2042, 2021. |
| [5] | A. Contreras-Vergara, S. Sifuentes-Franco, S. Haack, O. Graciano-Machuca, A. D. Rodriguez-Carrizalez, A. K. López-Contreras, I. V. Reyes-Pérez and S. G. Huerta-Olvera, "Impact of pharmaceutical education on medication adherence and its clinical efficacy in patients with type 2 diabetes and systemic arterial hypertension," *Patient preference and adherence,* p. 1999–2007, 2022. |
| [6] | M. Malik, A. Hussain, U. Aslam, A. Hashmi, M. Vaismoradi, K. Hayat and S. Jamshed, "Effectiveness of community pharmacy diabetes and hypertension care program: an unexplored opportunity for community pharmacists in Pakistan," *Frontiers in Pharmacology,* vol. 13, p. 710617, 2022. |
| [7] | W. Wang, L. Geng, C. Sun, H. Li, J. Wang and others, "Efficacy of pharmaceutical care in patients with type 2 diabetes mellitus and hypertension: a randomized controlled trial," *International Journal of Clinical Practice,* vol. 2022, 2022. |
| [8] | B. L. Carter, G. R. Bergus, J. D. Dawson, K. B. Farris, W. R. Doucette, E. A. Chrischilles and A. J. Hartz, "A cluster randomized trial to evaluate physician/pharmacist collaboration to improve blood pressure control," *The Journal of Clinical Hypertension,* vol. 10, p. 260–271, 2008. |
| [9] | D. L. McLean, F. A. McAlister, J. A. Johnson, K. M. King, M. J. Makowsky, C. A. Jones, R. T. Tsuyuki, S. C. R. I. P.-H. T. N. Investigators and others, "A randomized trial of the effect of community pharmacist and nurse care on improving blood pressure management in patients with diabetes mellitus: Study of Cardiovascular Risk Intervention by Pharmacists–Hypertension (SCRIP-HTN)," *Archives of internal medicine,* vol. 168, p. 2355–2361, 2008. |
| [10] | B. L. Carter, G. Ardery, J. D. Dawson, P. A. James, G. R. Bergus, W. R. Doucette, E. A. Chrischilles, C. L. Franciscus and Y. Xu, "Physician and pharmacist collaboration to improve blood pressure control," *Archives of internal medicine,* vol. 169, p. 1996–2002, 2009. |
| [11] | D. Edelman, S. K. Fredrickson, S. D. Melnyk, C. J. Coffman, A. S. Jeffreys, S. Datta, G. L. Jackson, A. C. Harris, N. S. Hamilton, H. Stewart and others, "Medical clinics versus usual care for patients with both diabetes and hypertension: a randomized trial," *Annals of internal medicine,* vol. 152, p. 689–696, 2010. |
| [12] | L. B. Cohen, T. H. Taveira, S. A. M. Khatana, A. G. Dooley, P. A. Pirraglia and W.-C. Wu, "Pharmacist-led shared medical appointments for multiple cardiovascular risk reduction in patients with type 2 diabetes," *The Diabetes Educator,* vol. 37, p. 801–812, 2011. |
| [13] | L. A. McGuinness and J. P. T. Higgins, "Risk-of-bias VISualization (robvis): An R package and Shiny web app for visualizing risk-of-bias assessments," *Research Synthesis Methods,* vol. n/a. |
| [14] | J. A. C. Sterne, J. Savović, M. J. Page, R. G. Elbers, N. S. Blencowe, I. Boutron, C. J. Cates, H.-Y. Cheng, M. S. Corbett, S. M. Eldridge and others, "RoB 2: a revised tool for assessing risk of bias in randomised trials," *bmj,* vol. 366, 2019. |
